# Spontaneous ovarian hyperstimulation syndrome with a previously undescribed gene mutation of the follicle-stimulating hormone receptor gene: a case report

**DOI:** 10.1016/j.xfre.2026.03.006

**Published:** 2026-04-28

**Authors:** Anne-Christin Loheit, Ekkehard Schleussner, Mike Fischer, Anna Kolterer

**Affiliations:** a Department of Obstetrics, University Hospital Jena, Am Klinikum 1, Friedrich Schiller University Jena, Jena, Germany; b Center for Early Pregnancy and Reproductive Health (CEPRE), Jena, Germany; c Institute of Human Genetics, University Hospital Jena, Am Klinikum 1, Friedrich Schiller University Jena, Jena, Germany

**Keywords:** Spontaneous ovarian hyperstimulation syndrome, FSHR gene, mutation, pregnancy

## Abstract

**Objective:**

To present a case of a patient with recurrent spontaneous ovarian hyperstimulation syndrome (sOHSS) and a previously undescribed mutation of the follicle-stimulating hormone receptor (FSHR).

**Design:**

Case report.

**Subjects:**

A 23-year-old pregnant woman (gravida II) with recurrent spontaneous ovarian hyperstimulation during 2 consecutive singleton pregnancies.

**Exposure:**

Spontaneous conception in the presence of a heterozygous FSHR gene mutation

**Main Outcome Measures:**

Clinical course of sOHSS, hormonal profile, sonographic findings, molecular genetic results, and maternal and fetal outcomes.

**Results:**

The patient presented at 12 weeks’ gestation with massively enlarged bilateral multicystic ovaries (‟kissing ovaries”) and mild ascites, without pleural effusion. Molecular genetic analysis revealed a previously unreported heterozygous FSHR mutation (c.1379T>A, p.Leu460Gln) in the transmembrane domain, classified as likely pathogenic according to American College of Medical Genetics and Genomics criteria. Conservative management resulted in gradual regression of ovarian size from the second trimester onward. The pregnancy was complicated by fetal growth restriction with normal uteroplacental and fetal perfusion parameters. Delivery by elective cesarean section at 38+0 weeks resulted in a healthy neonate.

**Conclusion:**

The discovery of a novel FSHR gene mutation, which is likely associated with sOHSS in this case, suggests that genetic factors may influence the development of this rare condition. It underlines the importance of considering genetic testing in managing and diagnosing sOHSS, potentially guiding more personalized and effective treatment strategies for affected women.

## Introduction

There are 2 known forms of ovarian hyperstimulation syndrome (OHSS): iatrogenic (iOHSS) in the context of assisted reproductive technology and spontaneous (sOHSS). Both forms are characterized by bilateral multiple cystic ovaries and a fluid shift into the extravascular space with intravascular volume depletion; depending on the severity, renal failure, and hypovolemic shock, and even death may occur (1). Iatrogenic OHSS develops as a result of the exogenous administration of gonadotropins to stimulate the follicles during fertility treatment. Human chorionic gonadotropin (hCG), which is used to induce ovulation, leads to the release of vasoactive substances from the ovaries, such as vascular endothelial growth factor. This increases the vascular permeability and, in severe cases, leads to the clinical picture of iOHSS. The severity of OHSS correlates with the degree of ovarian follicle formation (1). In contrast, sOHSS is rare and occurs after spontaneous conception. The symptoms usually appear around the 9th week of pregnancy with the physiological peak of placental hCG synthesis and disappear again after a few weeks (1).

Spontaneous OHSS can be divided into 2 subtypes. There can be an overstimulation of a normal follicle-stimulating hormone (FSH) receptor (FSHR) due to increased circulating levels of either hCG or thyroid-stimulating hormone (TSH). Increased hCG levels can be seen in molar or higher-grade multiple pregnancies (1, 2, 3). Moreover, increased TSH levels are found in TSH-producing pituitary adenoma or severe hypothyroidism. A second subtype of sOHSS is due to FSHR gene mutations that render the receptor abnormally sensitive to hCG or TSH, leading to ovarian hyperresponsiveness even at normal hormone levels (4).

**Figure 1:**
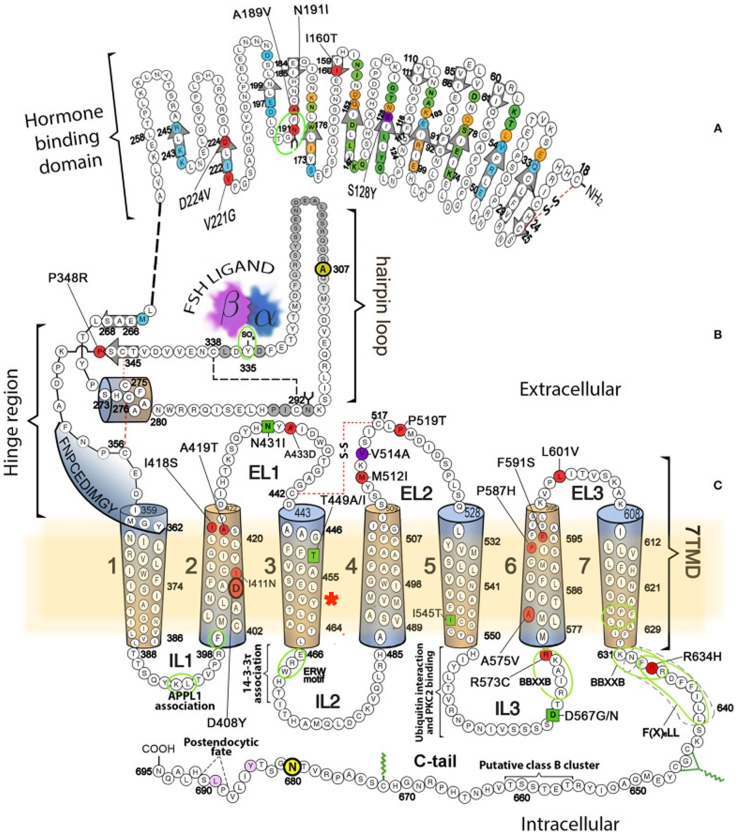
Structure of the follicle-stimulating hormone receptors (FSHRs) showing the hormone-binding domain, hinge region, 7 transmembrane segments, and the intracellular C terminus. New mutation at the *red asterisk*, known mutations to amino acids shaded in *red* lead to the FSHR’s loss of function; modified from Ulloa-Aguirre et al. (6) under the terms of the Creative Commons Attribution 4.0 license.

Various mutations in the FSHR gene, which encodes the FSHR, have been identified. (4, 5). Mutations have been described in both the transmembrane and extracellular domains of the receptor (3). The extracellular domain is responsible for the high affinity of hCG, whereas the transmembrane domain is primarily responsible for the transmission of the activity signal. Most mutations are located in the transmembrane domain (3). In this case report, we describe a novel mutation in the transmembrane domain of the FSHR with a sequence change c.1379T>A, p. (Leu460Gin) as a cause of sOHSS (Fig. 1) (6).

## Case report

**Figure 2:**
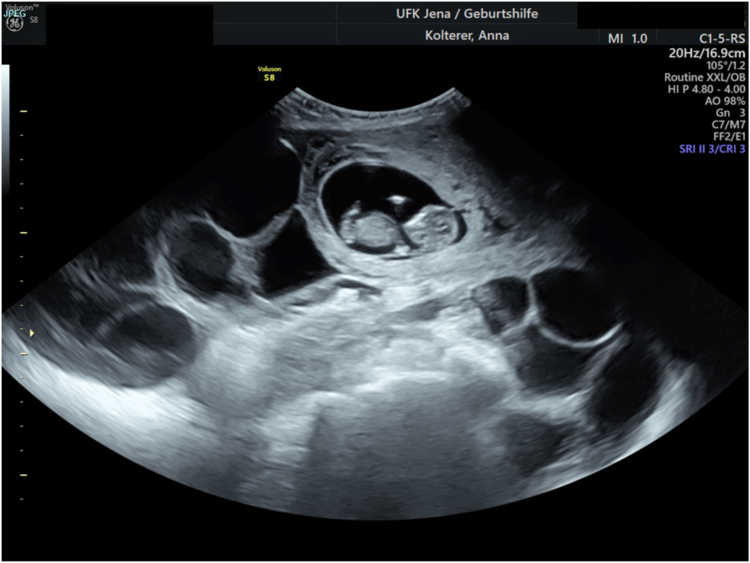
Enlarged extreme hyperstimulated ovaries with the intact pregnancy in between.

A 23-year-old pregnant woman (gravida II/para I) presented to our hospital at 12 weeks of spontaneous conception of a singleton pregnancy due to insufficient abdominal girth gain. Sonography revealed extremely hyperstimulated, up to 190 mm enlarged ‟kissing ovaries” (Fig. 2). Slight ascites but no pleural effusions could be demonstrated. No sonomorphological abnormalities were detected in the placenta or fetus, and they continued to develop normally. The patient reported similar complaints with enlarged ovaries and slight ascites in the previous pregnancy, but normal ovarian sonomorphology after the first pregnancy. The patient had a regular cycle between pregnancies. There is no information available on the number of corpora lutea. The patient became pregnant spontaneously in both pregnancies. The hCG level was measured in a high-normal range for the 12th week (118.133 mIU/mL) (7), whereas luteinizing hormone and FSH were physiologically suppressed by gestation (Table 1). Hyperprolactinemia and nonphysiological TSH concentrations could be excluded (Table 1). The initial and highest hematocrit (Hkt) measured was 40%, with a hemoglobin level of 7.9 mmol/L. Given the patient’s history of possible recurrent sOHSS in 2 consecutive pregnancies, molecular genetic analysis of FSHR abnormalities was conducted on maternal ethylenediaminetetraacetic acid blood after informed consent. Medical care was initially provided on an outpatient basis against medical advice. In addition to daily checks of Hkt values, body weight, and abdominal circumference, thrombosis prophylaxis with low-molecular-weight heparin and intravenous fluid therapy were administered depending on the Hkt (target value <35%, fluid 1-2l/d). At 13 weeks’ gestation, the patient’s abdominal circumference increased from 81 cm to 84.5 cm, accompanied by enlargement of the left ovary, for which she was hospitalized for several days. Laboratory tests showed no hemoconcentration (Hkt 34%, hemoglobin 6.9 mmol/L). In a stable general condition and with regressive sonographic findings, the patient was discharged after 5 days. Beginning in the 14th week, the ovaries started to shrink, and their size was reduced to 52 mm by the 25th week. During the pregnancy, a fetal growth restriction below the 3rd percentile was identified, with normal uteroplacental and fetal perfusion parameters. An elective cesarean section was performed at 38+0 weeks after fetal growth trajectories within this low percentile (birth weight 2775g > 10th percentile); appearance, pulse, grimace, activity, and respiration score 8/9/9; pH_art_ 7.29, base excess −0.3). The patient has consented to publication.

**Table 1:** Hormone levels during pregnancy.

	11+0^a^	11+1^a^	11+6^a^	34+2^a^	38+4^a^
hCG (mIU/mL)	118.133			74.533	18.091
LH (mIU/mL)		0.3			
FSH (mIU/mL)		0.3			
Prolactin (ng/mL)		55.28			
TSH (mIU/L)	3.07				
fT3 (pg/mL)		3.63			
fT4 (pg/mL)		9.44			
Estradiol (pg/mL)			16.070		
Progesterone (ng/mL)			129.245		

*Note:* FSH = follicle-stimulating hormone; fT3 = free triiodothyronin; fT4 = free thyroxine; hCG = human chorionic gonadotropin; LH = luteinizing hormone; TSH = thyroid-stimulating hormone.

a Week of pregnancy.

## Discussion

In the pathophysiology of sOHSS, a molar pregnancy, ovarian tumors, and pituitary adenomas have been considered as possible causal contributors. In this case, it could be ruled out on the basis of the normal sonomorphology of the placenta and the absence of markedly elevated hCG levels and normal TSH values. Through interdisciplinary counseling, the patient agreed to molecular genetic diagnostics, which identified a previously unreported heterozygous mutation in the FSHR gene. The sequence change c.1379T>A, p. (Leu460Gin) leads to a replacement of the amino acid leucine by glutamine at position 460 and was classified as likely pathogenic according to the American College of Medical Genetics and Genomics criteria. This change has not yet been demonstrated in the general population, and no data or functional studies are currently available. However, the in-silico prediction algorithms queried consistently classify the protein effect as deleterious, and the affected gene region is known to be a hotspot for pathogenic variants. In addition, the patient’s phenotype was highly specific for a functional defect of the FSH receptor gene. Zhang et al. (8) showed that mutations at position L460 in both the luteinizing hormone receptor and FSHR genes lead to increased baseline activity of the luteinizing hormone and FSH receptors. If leucine at position L460 is replaced by lysine, arginine, alanine, or asparagine, the receptor only shows increased activity with arginine (9). No cause for the fetal growth restriction could be detected. In summary, it can be said that the connection between the genetic defect and the patient’s constellation of symptoms is very likely, but functional analyses are necessary for a final assessment of the genetic defect and its phenotypic expression. To date, there is no evidence demonstrating an association between sOHSS and fetal growth restriction. A few studies have investigated the relationship between iOHSS and fetal growth restriction, with inconsistent findings (10, 11).

## Conclusion

sOHSS should be considered as a differential diagnosis in patients with isolated enlarged hyperstimulated ovaries during pregnancy. In contrast to our patient, previous case reports have described marked ascites or pleural effusions. (12). In the case of sOHSS, molecular genetic analysis of FSHR mutations should be considered. Women with FSHR mutations should be monitored closely to avoid complications in subsequent pregnancies.

### Declaration of Generative AI and AI-Assisted Technologies in the Writing Process

During the preparation of this work, the authors used ChatGPT to correct grammatical errors. After using this tool/service, the authors reviewed and edited the content as needed and take full responsibility for the content of the publication.

## CRediT Authorship Contribution Statement

**Anne-Christin Loheit:** Writing – original draft, Visualization, Methodology, Investigation, Funding acquisition, Formal analysis, Conceptualization. **Ekkehard Schleussner:** Writing – review & editing, Visualization, Investigation. **Mike Fischer:** Writing – review & editing, Methodology, Investigation. **Anna Kolterer:** Writing – review & editing, Visualization, Methodology, Investigation, Conceptualization.

## Declaration of Interests

A-C.L. has nothing to disclose. E.S. has nothing to disclose. M.F. has nothing to disclose. A.K. has nothing to disclose.
